# Short-Term Surgical Outcomes of Curative Colorectal Resections from an Evolving Low-Volume Cancer Center in a Tier-2 City in India

**DOI:** 10.1007/s13193-025-02339-z

**Published:** 2025-05-26

**Authors:** Vishnu S. Menon, Amita Sekhar Padhy, Rigved Nittala, Mounika Basani, Sidaksingh R. Arora

**Affiliations:** Department of Surgical Oncology, Homi Bhabha Cancer Hospital & Research Centre, Visakhapatnam, 530053 Andhra Pradesh India

**Keywords:** Colon cancer, Rectal cancer, CRC, Surgical outcomes

## Abstract

Colorectal cancers (CRC) are the fourth most prevalent cancer in India. Treatment modalities range from surgery, chemotherapy, radiotherapy, targeted treatment, and immunotherapy, with surgery forming the cornerstone of curative treatment in combination with any of the above. We sought to explore the short-term surgical outcomes of curative colorectal resections from our center and compare them with the published outcomes elsewhere. This is a retrospective study of all colorectal cancers that underwent curative resections at our center, from 1st January 2017 to 31st October 2024. Patients were identified from a prospectively maintained surgical database and electronic medical records. The clinical, radiological, histopathological features, and 30-day surgical outcomes were evaluated. We identified 207 patients for the said duration, with the majority of them being males (60.9%, 126/207), left-sided tumors (70%, 145/207) and clinic-radiologically stage III cancers (66.2%, 137/207). Preoperative treatment was employed in 38.7% (80/207) patients who were mostly rectal primaries (78/80). A minimally invasive surgical (MIS) approach was attempted in 36 patients with a conversion rate of 16.7%. Extended resections were performed in 33 patients (15.9%). The median length of hospital stay was 7 days (range 5 to 34 days). We observed re-exploration rates of 7.2%, a readmission rate of 3.4%, major perioperative morbidity (Clavien-Dindo 3a or above) of 13%, and 30-day perioperative mortality of 2.9%. Margin-negative resections were achievable in almost all cases (99.5%, 206/207), and optimal nodal yield (12 or more) was attained in 90.8% (188/207). Our study provides preliminary evidence that safe colorectal resections, including extended resections, can be performed in low-volume and resource-constrained centers with acceptable perioperative morbidity.

## Introduction

In India, colorectal cancers (CRC) are the sixth most common cancer by the number of new cases and, overall, the fourth most prevalent cancer [1]. While CRC accounts for 4.5% of all cancer-related deaths in India, the reported 5-year overall survival for the same is among the lowest in the world [1, 2]. In India, on one side, we grapple with the resource constraints of being a largely agrarian economy with limited health care resources; on the other side, we are facing a high prevalence of CRC with changing lifestyles and dietary habits in an increasingly urban populace [3]. This, along with the sheer geographical size and lack of uniform reporting of outcomes of colorectal cancer across the country, makes it a challenge to identify the lacunae in our existing setup and poses difficulty for the government health machinery to allocate resources [4]. The treatment landscape for CRC is rapidly evolving with ever-changing role of treatment modalities  including surgery, chemotherapy, immunotherapy, and radiotherapy [5]. Surgery forms the crux of curative treatment of CRC, with the same being employed in 60% of all cases [6]. Other pathologies, like neuroendocrine tumors, melanoma, and gastrointestinal stromal tumors, are being more readily diagnosed, and the surgical principles are similar to CRC(adenocarcinoma) resections. Our center, which is located in a Tier-2 city in Eastern India, manages a spectrum of cancer cases, and a dedicated colorectal unit was not present until recently. In this study, we seek to audit the short-term surgical outcomes of all colorectal resections from our center. We aim to identify the challenges we face as a low-volume colorectal center, which possibly could serve as a mirror for similar setups across the country.

## Methods

### Study Design, Population, and Exclusion Criteria

This study was a retrospective cohort analysis conducted at the colorectal surgical unit of a tertiary cancer center in India. A prospectively maintained institutional surgical database and the electronic medical records (EMR), between 1 st January 2017 and 31 st October 2024, were accessed to identify patients who underwent curative colorectal resections. The following inclusion criteria were applied: (1) colorectal resections for malignancies including colorectal adenocarcinoma, neuroendocrine tumors, melanoma; (2) underwent curative resection during the said duration. The following exclusion criteria were applied: (1) recurrent disease, (2) colorectal resections for benign pathology.

### Data Retrieval and Defining Endpoints

Clinical information, including patient demographics, pathological characteristics, surgical details, postoperative outcomes, chemotherapy, and radiation (neo/adjuvant), was collected from the surgical database and EMR. The extent of the disease was confirmed with preoperative imaging, operative notes, and histopathology reports. Patients with missing datasets for a particular variable were recorded as missing. The colorectal disease was divided based on sidedness, with the right-sided CRC tumors arising from the ascending colon and proximal two-thirds of the transverse colon and the left-sided CRC tumors arising from the rectum, descending and sigmoid colon, and distal one-third of the transverse colon. The primary endpoint of this study was to evaluate the short-term surgical outcomes of all curative colorectal resections at our center. The secondary endpoint was to compare the short-term outcomes at our center with published outcomes from other low-volume and high-volume centers.

### Statistical Analysis

Descriptive statistics were used to analyze clinical, pathological, and radiological features and management protocols. Data was analyzed with IBM SPSS version 29 [7]. Categorical.

variables were presented as numbers with percentages and were compared among groups using chi-square *t* test and Fisher’s exact test when needed. The distribution of continuous variables was reported as median with range and was compared using the Mann–Whitney *U* test. We took a *p* value of 0.05 or less as significant.

## Results

### *Cohort Characteristics (*Table 1*)*

**Table 1:** Cohort characteristics

	Median (range)	Mean +/− standard deviation
**Age (in years)**	60 (22–85)	58.7 +/− 13.6
**Hemoglobin (in grams per deciliter)**	11.0 (7.8–14.0)	11.4 +/− 4.3
**Serum creatinine (in milligram per deciliter)**	0.9 (0.5–1.78)	0.95 +/− 0.69
**Body mass index (in kilogram per meter square)**	23.5 (18.5–32)	24.1 +/− 7.8
	**Frequency**	**Percentage**
**Sex**		
Male	126	60.9
Females	81	39.1
**Sites**		
Right sided	52	25.1
Left sided	145	70
Both sides	10	4.8
**Stage at presentation**		
Early (stage I and II)	59	28.5
Locally advanced (stage III)	137	66.2
Metastatic (stage IV)	11	5.3
**American Society of Anesthesiologist (ASA) grade for preoperative health status**		
ASA1 or 2	192	92.8
ASA3 or above	15	7.2
**Preoperative treatment**		
Yes	80	38.6
Neoadjuvant chemotherapy	2	9.6
Neoadjuvant chemo-radiotherapy	76	36.7
Total Neoadjuvant therapy	2	9.6
No (upfront)	127	61.3
Emergency	19	9.1
**Surgical procedures**		
Right hemicolectomy	36	17.4
Right extended Hemicolectomy	20	9.7
Left hemicolectomy	9	4.3
Sigmoidectomy	12	5.8
Anterior resection	65	31.4
Low anterior resection	5	2.4
Abdominoperineal resection	49	23.7
Total procto-colectomy	9	4.4
Posterior exenteration	2	1
**Extended resections**		
No	174	84
Yes	33	16
Groin node dissection	3	1.4
Pelvic lymph node dissection	5	2.4
Retroperitoneal lymph node dissection	1	0.48
Vaginal resections	2	1
Oophorectomy	3	1.4
Hysterectomy	3	1.4
Prostate shave	1	0.48
Seminal vesicle resection	1	0.48
Urinary bladder	4	1.9
Coccyx	1	0.48
Small bowel resection	1	0.48
Abdominal wall resections	2	1
Peritoneal cytoreductions	3	1.4
Psoas muscle	1	0.48
Liver resections	2	1
**Histopathology**		
Adenocarcinoma	200	96.6
Melanoma	5	2.4
Neuroendocrine cancers	2	1
**Resection margins**		
R0	206	99.5
R1/R2	1	0.5
**Nodal yield**		
12 or more lymph nodes	188	90.8
Less than 12 lymph nodes	19	9.2
**Short-term outcomes**		
Clavien-Dindo Grade 2 or below	180	87
30 days morbidity (Clavien-Dindo Grade 3 or above)	27	13
30 days mortality rate	6	2.9
Re-exploration rate	15	7.2
Readmission rate	7	3.4
Perioperative blood transfusion	44	21.3
Surgical site infection	19	9.2
Prolonged antibiotic usage	37	17.9
Anastomotic leak rate	4	1.9
	*Median (range)*	*Mean* + */− SD*
Median blood loss (in milliliters)	450 (100–3000)	500. 7 +/− 244.9
Duration of surgery (in minutes)	240 (180–450)	255 +/− 77.8
Duration of urinary diversion (in days)	2 (1–28)	3.36 +/− 3.8
Time to passage of flatulence (in days)	1 (1–9)	1.59 +/− 1.12
Length of hospital stay, LOHS (in days)	7 (5–34)	7.87 +/− 3.68

We identified 207 patients who had undergone curative colorectal resections for the said duration, with the majority of them being males (60.9%, 126/207), left-sided tumors (70%, 145/207), and clinic-radiologically stage III cancers (66.2%, 137/207). Preoperative treatment was employed in 80/207 patients, mostly rectal primaries, with neoadjuvant chemoradiation being used in 78/80 patients with two of whom required total neoadjuvant treatment, and 68/78 of them receiving long-course chemoradiation (Table 1). Upfront surgery was offered in 127/207 patients (61.3%) with 86/127 (67.7%) being colonic primaries.

### Perioperative Course (Table 1)

The median duration of surgery was 240 min (180–450 min), and median blood loss was 450 ml (100–3000 ml). Minimally invasive surgical approach (MIS), i.e., laparoscopy, was attempted in 36 patients with a conversion rate of 16.7%. Perioperative blood transfusions were required in 21.3% of cases (44/207). The median time of urinary diversion with a Foley catheter was 2 days, ranging from 1 to 28 days. Four patients who required urinary diversion for more than 21 days had all undergone some form of urinary bladder resections (Tables 1 and 2). The median length of hospital stay was 7 days, ranging from 5 to 34 days. The 30-day perioperative morbidity with Clavien-Dindo Grade 3 or above was observed in 13.5% of patients (27/207). We had a re-exploration rate of 7.2% (15/207) with a third of them being stoma-related complications. We observed prolonged antibiotic requirements in 37/207 patients, with 19/37 being for surgical site infections. The anastomotic leak rate was observed in 4 patients, and all could be salvaged. We observed a 30-day mortality rate of 2.9% (6/207) with medical complications contributing to half of them. Margin-negative resections were achievable in almost all cases (99.5%, 206/207), and optimal nodal yield (12 or more) was attained in 90.8% (188/207).

**Table 2:** Short term outcomes described site-wise

Parameters		Overall		Colon		Rectum		Synchronous		*p* vale
Total number of cases		207 (100)		86 (41.5)		111 (53.6)		10 (4.8)		
Age (in years)	*Median (range)*	60 (22–85)		62 (24–83)		60 (22–85)		50.5 (33–80)		0.277
	*Mean*	58.8 +/− 13.6		58.3 +/− 14.2		59.6 +/− 13.1		52.7 +/− 13.10		
Sex	*Males*	126 (60.9)		48 (55.8)		70 (63.1)		2 (20)		0.261
	*Females*	81 (39.1)		38 (44.2)		41 (36.9)		8 (80)		
Preoperative treatment	*Yes*	80 (28.6)		3 (3.5)		76 (68.5)		1 (10)		< 0.001
T-stage	*T1*	19 (9.2)		0(0)		17 (15.3)		2 (20)		< 0.01
	*T2*	30 (14.5)		10 (11.6)		20 (18)		0 (0)		
	*T3*	122 (58.9)		47 (54.7)		68 (61.3)		7 (70)		
	*T4*	36 (17.4)		29 (33.7)		66 (5.4)		1 (10)		
N stage	*N0*	108 (52.2)		36 (41.9)		67 (60.4)		5 (50)		0.035
	*N* +	99 (47.8)		50 (58.1)		44 (39.6)		5 (50)		
M stage	*M0*	196 (94.7)		200 (91.9)		203 (96.4)		10 (100)		0.002
	*M* +	11 (5.3)		7 (8.1)		4 (3.6)		0 (0)		
Approach	*Open*	177 (85.5)		80 (93)		88 (79.3)		9 (90)		0.023
	*Laparoscopic*	30 (14.5)		6 (7)		23 (20.7)		1 (10)		
Extended resection	*yes*	33 (15.9)		16 (18.6)		17 (15.3)		0 (0)		0.307
Perioperative blood transfusion	*yes*	44 (21.3)		17 (19.8)		23 (20.7)		4 (40)		0.331
Surgical site infection	*Yes*	19 (9.2)		10 (11.6)		7 (6.3)		2 (20)		0.212
Prolonged antibiotic usage	*Yes*	37 (17.9)		13 (15.1)		23 (20.7)		1 (10)		0.481
Anastomotic leak		4 (1.9)		0 (0)		4 (3.6)		0 (0)		0.173
90-day morbidity		28 (13.5)		8 (9.3)		19 (17.1)		1 (10)		0.270
Failure to rescue		6 (2.9)		2 (2.3)		3 (2.7)		1 (2)		0.224
Re-exploration		15 (7.2)		1 (1.2)		14 (12.6)		0(0)		0.006
Readmission rate		7 (3.4)		2 (2.3)		5 (4.5)		0 (0)		0.585
		*Median (Range)*	*Mean* + */− SD*	*Median (Range)*	*Mean* + */− SD*	*Median (Range)*	*Mean* + */− SD*	*Median (Range)*	*Mean* + */− SD*	
Median blood loss	*In mL*	450 (100–3000)	500. 7 +/− 244.9	450 (100–1500)	488.9 +/− 178.8	450 (200–3000)	513.7 +/− 294.0	435 (400–700)	479.6 +/− 103.7	0.750
Duration of surgery	*In minutes*	240 (180–450)	255 +/− 77.8	240 (180–450)	254.0 +/− 80.4	240 (180–240)	257.6 +/− 75.2	200 (180–410)	242 +/− 90.8	0.832
Duration of urinary diversion	*In days*	2 (1–28)	3.36 +/− 3.8	2 (1–28)	3.10 +/− 3.4	2 (1–24)	3.6 +/− 4.3	2 (2–6)	2.9 +/− 1.37	0.660
Time to passage of flatulence	*In days*	1 (1–9)	1.59 +/− 1.12	1 (1–9)	1.43 +/− 1.10	1 (1–6)	1.75 +/− 1.08	1 (1–6)	1.5 +/− 1.58	0.174
Length of hospital stay, LOHS	*In days*	7 (5–34)	7.87 +/− 3.68	7 (5–20)	7.8 +/− 3	7 (5–34)	7.95 +/− 4.27	7 (6–10)	7.5 +/− 1.50	0.919
Number of cases per year		23 (7–51)	25.8 +/− 14.6	11 (4–16)	10.8 +/− 4.5	10.5 (2–33)	13.87 +/− 10.48	1.5 (1–3)	1.67 +/− 0.81	–

### Comparing Outcomes in Colonic and Rectal Resections (Tables 1, 2)

The outcomes in the colonic and rectal subsets were comparable in certain parameters, with similar median duration of surgery, blood loss, duration of urinary diversion, and time to passage of flatulence (Table 2). However, on further comparing rectal cancer surgeries with colonic surgery, a higher proportion of major perioperative morbidity (17.1% versus 9.3%), re-exploration rates (12.6% versus 1.2%), and surgical mortality (2.7% versus 2.3%) was noted, but all were statistically not significant. We operated on 10 patients with synchronous rectal and colonic primaries, and their outcomes were comparable with both the colon only and rectal only subsets.

### Tryst with Minimally Invasive and Extended Resections (Fig. 1)

**Fig. 1:**
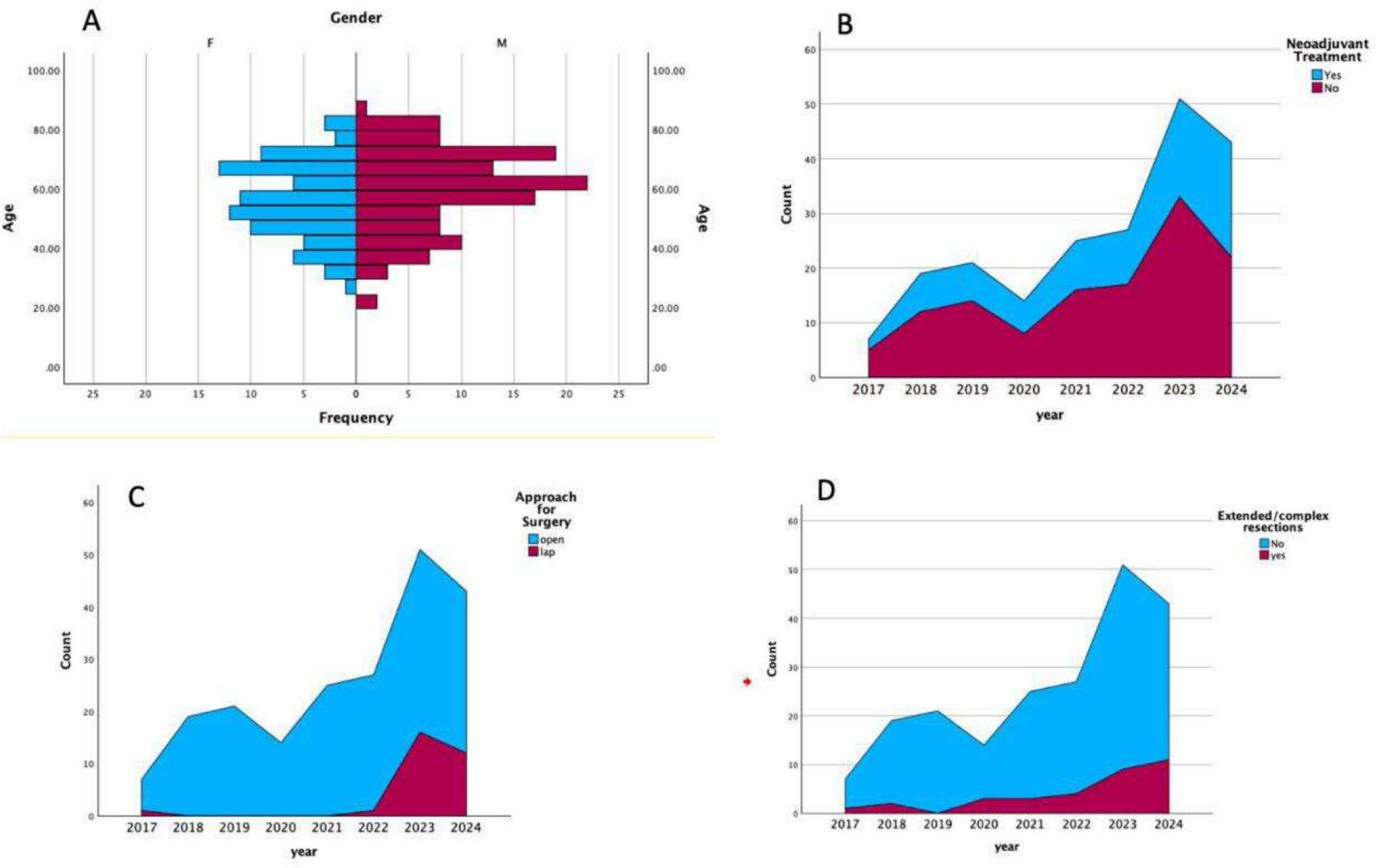
A Population Age pyramid of the cohort split by sex (Gender) of patients. B Area charts based on the preoperative treatment. C Area Charts based on the surgical approach. D Area charts representing the extended/complex resections performed

We Observed one minimally invasive procedure and 6 extended resections in the period between 2017 and 2020, while the same had risen to 29 and 27, respectively, in the 2021–2024 period. This could partly be due to developments that we saw as an oncology designated center, with the start of dedicated modular operative rooms, increasing patient footfall, and the start of interdisciplinary tumor boards from 2020 onwards, as well as the evolution of the surgeons and OT nursing team. Successful minimally invasive colorectal resections were feasible in 30 patients, and the median blood loss, postoperative urinary diversion, time to passage of flatulence, and length of hospital stay were lesser compared to open cases (Table 3). We observed similar short-term outcomes between laparoscopic and open subsets, with similar nodal yield, margin status, and perioperative morbidity (Table 3). The median duration of MIS surgery had remained similar over the course of the past 2 years, despite the learning curve achieved by the senior surgeons, which could be primarily attributed to the nature of the functioning of our unit, with several short-stay surgical trainees also performing certain steps under supervision. Extended resection was performed in 33 patients that included multivisceral resections in 19 patients, extended lymph node dissection in 9 patients, and abdominal wall/peritoneal cytoreduction that was required in 5 patients.

**Table 3:** Short term outcomes based on surgical approach

Parameters	Overall		Laparoscopic		Open		*p* value
	*Frequency (percentage)*		*Frequency (percentage)*		*Frequency (percentage)*		
Sidedness	207		30 (14.5)		177 (85.5)		0.116
Left	145 (70)		25 (83.3)		120 (67.8)		_
Right	52 (25.1)		4 (13.3)		48 (27.1)		
Both	10 (4.8)		1 (3.3)		9 (5.1)		
Time period							< 0.001
*2017 to 2020*	61 (29.5)		1 (1.6)		60 (98.4)		
*2021 to 2024*	146 (70.5)		29 (19.8)		117 (80.1)		
Perioperative blood transfusion	44 (21.3)		1 (3.3)		43 (24.3)		0.009
Surgical site infection	19 (9.2)		2 (6.7)		17 (9.6)		0.608
Prolonged antibiotic usage	37 (17.9)		6 (20)		31 (17.5)		0.372
Anastomotic leak rate	4 (1.9)		1 (3.3)		3 (1.7)		0.32
Positive margin status	1 (0.48)		0 (0)		1 (0.56)		0.51
	*Median (Range)*	*Mean* + */− SD*	*Median (Range)*	*Mean* + */− SD*	*Median (Range)*	*Mean* + */− SD*	
Median blood loss (in mL)	450 (100–3000)	500.7 +/− 244.9	340 (100–600)	350 +/− 114.5	450 (400–3000)	526 +/− 252.3	< 0.001
Duration of surgery (in minutes)	240 (180–450)	255 +/− 77.8	280 (180–400)	291 +/− 74	240 (180–450)	249 +/− 77	0.003
Duration of urinary diversion (in days)	2 (1–28)	3.36 +/− 3.8	1 (1–24)	3.13 +/− 4.9	2 (2–28)	3.4 +/− 3.6	0.388
Time to passage of flatulence (in days)	1 (1–9)	1.59 +/− 1.12	1 (1–4)	1.35 +/− 0.67	1 (1–9)	1.63 +/− 1.18	0.043
Length of hospital stay, LOHS (in days)	7 (5–34)	7.87 +/− 3.68	6 (5–28)	7.3 +/− 4.28	7 (5–34)	7.97 +/− 3.57	0.209
Lymph node yield	24 (0–132)	27.72 +/− 17.52	22 (4–116)	25.6 +/− 20.90	25 (0–132)	28.08 +/− 16.92	0.108

### Testing Times During COVID19 Pandemic

During the duration between 1 st January 2020 and 30 th June 2021, i.e., active COVID era, a total of 28 were operated on. Mitigation strategies, including segregation of care pathways with COVID and non-COVID zones, preoperative COVID19 testing, less frequent follow-up imaging, and implementation of a hybrid model of tumor board discussions, were employed to curtail the impact of the pandemic. Though there was minimal disruption of healthcare machinery at our center, there was marked disruption in the minimally invasive procedures, as none of the patients underwent the same during this period, which could have been due to the hospital policy to limit aerosolization during laparoscopic procedures as much as feasible. We observed a 30-day major morbidity rate of 17.9% (5 out of 28), but there were no perioperative deaths. The median length of hospital stay was 7 days (ranging from 6 to 34 days) which was similar to the overall cohort.

## Discussion

The main objective of this study was to study the short-term surgical outcome for curative colorectal resections from our center. The literature pertaining to the short-term outcomes from such a setup from our region, i.e., the eastern part of the Indian subcontinent, is scanty. We observed the median number of colectomies, rectal surgeries, and overall colorectal resections per year (2021–2024) as 11, 10.5, and 23, respectively, ie 70.5% (146/207) of the total cohort. Firstly, to answer the question of whether we qualify as low-volume or a high-volume center, we do not have any published literature from our country for such classification. Furthermore, surgical volume-based categorization for colorectal units from elsewhere, based on the number of surgeries, is also wide-ranging, with some defining it based on the individual surgeon’s volume or hospital volume and some literature defining volume for colon and rectal surgeries separately [8, 9, 10, 11]. On using Damle et al. definition for colonic resections, we stand as a medium-volume center (9–18 per year) though we are at the cusp of transitioning to a high-volume center (18 or more per year) [9]. The German Cancer society (DKG) defined high volume as more than 40 colonic resections, which is far more than the number of cases we are currently performing; hence, we remain a low-volume center by this definition [12]. In the context of rectal primaries, 20 or more resections are defined as the number to categorize a high volume, and our median yearly output was 11 cases per year [10]. Overall, the definition for high volume for colorectal resections was wide ranging, with the American colorectal society suggesting 21 cases per year and the Dutch definition being 50 cases per year [12, 13]. Although with respect to the number of cases we operate, we are a low-volume center, we are transitioning to a high volume with increasing complexity of cases and challenges associated with them. Apart from this, the number of complex colorectal resections is also on the rise at our center, which poses unique challenges to carefully select patients and the surgical skills to perform the same [14].

Secondly, with regard to where we stand in terms of short-term surgical outcomes when compared to the published outcomes, we compared our outcomes both with low- and high-volume centers (Table 4). On average, our duration of surgeries was longer compared to Tan et al. and Yılmaz et al. (Table 4) [15, 16]. But it is important to note that we are a training center with surgical trainees also performing surgeries under the guidance of senior surgeons to ensure quality training without compromising patient safety. In the pathological quality of surgical resections indices, our margin-positive resection status and suboptimal nodal yield rates were lower than the results published by Warps et al., Siraguasa et al., and Rottoli et al., but not comparable to the results of Damle et al. [8, 9, 17, 18]. Readmission after discharge, which could be considered a surrogate for poor postoperative recovery and increased health care expenditure, was 3.4% in our cohort. While this number is lower than the results in the Dutch Colorectal audit (7.5% for colon and 13.8% for rectum), it is nevertheless a number we need to improve [17]. Our perioperative mortality was 2.9% [6 cases] of which three were due to medical complications. Our major short-term morbidity, defined as Clavien-Dindo Grade 3 or above, was higher in the rectal patients than in colonic patients (Table 2). But it is important to understand that the treatment approach is different for both subsets, and our outcomes were similar to the ones published by the Dutch colorectal audit [17]. Based on these results (Table 4), we could achieve safe oncological resection and ensure reasonably good short-term surgical outcomes comparable to that of other low-volume and high-volume centers [8, 9, 15, 16, 17, 18, 19].

**Table 4:** Comparing short term outcomes with published literature

Parameters	Our study (2024)	Dutch colorectal audit (2021)^17^		Tan et al.^15^	Yılmaz et al. (2016)^16^	Göksoy et al. (2020^19^	Siragusa et al. (2021)^18^	COVID-CRC Study Group (2024)^8^		Damle et al. (2021)^9^		
		Colon cancer	Rectal cancer	Low volume	Low volume	Low volume	low volume	Low Volume (< 10/yr)	High Volume (> = 10/yr)	low (< 5/yr)	medium (5–11/yr)	High (> 11/yr)
Number of patients	207	68,833	28,546	23	30	20	86	999	4675	2127	3753	3540
Duration of surgery (in minutes)	255 +/− 77.8	NA	NA	138.35 +/− 45.96	149.67 +/− 39.21	172 ± 31	190 +/− 63	NA	NA	NA	NA	NA
Blood loss (in mL)	500. 7 +/− 244.9	NA	NA	NA	NA	NA	NA	NA	NA	NA	NA	NA
LOHS	7.87 +/− 3.68	NA	NA	6.26 +/− 3.41	6.87 +/− 6.09	7 ± 3	6.5 +/− 3.8	Median = 8	Median = 7	6 +/− 7	6 +/− 7	5 +/− 9
LOHS > 14 days	14 (6.8%)	14%	17.20%	NA	NA	NA	NA	NA	NA	NA	NA	NA
Time to passage of flatulence (in days)	1.59 +/− 1.12	NA	NA	NA		2 ± 0.7	NA	NA	NA	NA	NA	NA
Duration of urinary diversion (in days)	3.36 +/− 3.8	NA	NA	NA	NA	NA	NA	NA	NA	NA	NA	NA
Surgical site Infection	19 (9.2%)	NA	NA	2 (8.7)	5 (16.7)	3 (15)	6%	5%	3.80%	NA	NA	NA
Anastomotic leak rate	4 (1.9%)	NA	NA	2 (8.7)	2 (6.7)	NA	NA	3.40%	1.70%	NA	NA	NA
Readmission rate	7 (3.4%)	7.50%	13.80%	NA	NA	NA	2–2.3%	NA	NA	NA	NA	NA
Re-exploration rate	15 (7.2%)	8.60%	12%	NA	NA	NA	3–3.5%	NA	NA	4%	3%	2%
R1/R2 resection margins	1 (0.5%)	2.30%	6.40%	NA	NA	NA	4–4.7%	2%	2.30%	NA	NA	NA
nodal yield < 12	19 (9.2%)	20.10%	31.70%	NA	NA	NA	NA	17.40%	13.30%	NA	NA	NA
Perioperative morbidity (Clavien-Dindo Grade 3 or above)	28 (13.5)	16%	20.10%	NA	NA	NA	9.20%	19.50%	14.90%	8%%	8%	7%
Failure to rescue	6 (2.9%)	10.40%		NA	NA	NA	1–1.2%	2.50%	1.50%	NA	NA	NA

### Our Experience at Homi Bhabha Cancer Hospital & Research Centre, Visakhapatnam

Ours is a tertiary cancer center under the Tata Memorial Centre (Mumbai) under the Department of Atomic Energy, Government of India, and this larger association has helped in pushing for standardization of certain protocols, particularly in terms of preoperative treatment planning. However, the limited access to resources, particularly due to the remote location and limited man-power, there are certain challenges like the limited availability of mechanical sutures, energy devices, and laparoscopy-trained nursing staff. Also, implementation of *Enhanced* *Recovery after Surgery* (ERAS) fast-track principles is being addressed now with active multidisciplinary collaborations. At our center, interdisciplinary team discussion for selection of patients and close coordination between surgeons, anaesthesiologists, nursing team, physiotherapists, dieticians, ostomy nurse, and the patient’s guardian, with the patient at the core in the perioperative period, have become the cornerstone of the surgical management. With more accessibility to our center, growing experience of the surgical team, and the increasing number of cases and greater governmental support for covering treatment costs, we are expecting improved perioperative outcomes. Also, the standardization of preoperative evaluation and surgical steps and monthly surgical audits have actively contributed to our pursuit of improving the outcomes [20, 21].

### Strengths and Limitations

A major strength of this study is that it provides real-world data about short-term surgical outcomes in a Tier-2 city in India and the evolution of the colorectal unit at our center. However, the limitations of the study also need to be addressed, which include its retrospective nature, single-institution data, and missingness of datasets for many past patients. Further, the long-term survival outcomes were not studied in our cohort, as the majority of our patients were operated on in the last 2 years. Hence, clinically meaningful follow-up was not achieved to calculate the survival outcomes. Also, the study includes other pathologies, including melanoma and neuroendocrine tumors, and it is important to note that the focus of this study is evaluating short-term surgical outcomes of curative colorectal resections and is not restricted to colorectal adenocarcinoma alone.

### Looking Forward

Through the National Cancer Grid of India, it is the right time for member institutions to collaborate and develop India-centric registries, identify challengesand propose India-centric solutions. This can help upcoming centers like ours to compare their outcomes and evolve methods to take corrective measures [22]. In the long run, this can help in re-allocation of resources to centers across India and develop centers of excellence for colorectal cancer treatment and cater to the needs and challenges unique to each region.

## Conclusion

Our study provides preliminary evidence that safe colorectal resections with acceptable perioperative morbidity can be achieved in low-volume and resource-constrained centers. Nationwide consensus building for defining surgical volume and standard reporting of short-term surgical outcomes for colorectal resections needs to be encouraged.

## Conflict of interest

The authors declare no competing interests.

## Data Availability

Data is available on reasonable request from the corresponding author.
